# Correction: The Fabrication of Nano-Particles in aqueous solution from Oxyfluoride Glass Ceramics by Thermal Induction and Corrosion Treatment

**DOI:** 10.1186/1556-276X-6-509

**Published:** 2011-08-25

**Authors:** Hua Yu, Nan Hu, Ya-Nan Wang, Zi-Lan Wang, Zong-Song Gan, Li-Juan Zhao

**Affiliations:** 1 Photonics Center, College of Physical Science, Nankai University, Tianjin, 300071, China; 2 Tianjin Key Lab of Photonics Material and Technology for Information Science and The Key Lab of Weak Light Nonlinear Photonics, Ministry of Education, Nankai University, Tianjin, 300457, China

## Correction

The key reference to work of Mortier and Patriarche had been inadvertently omitted from this article [1]. The statement below in the original INTRODUCTION section is not very accurate and appropriate:

"Unfortunately, ever since the first report on oxyfluoride GCs, there have been much research on the properties of nano-particles in GCs but no publication has been reported about how to obtain free nano-particles in aqueous solution from the GC-host and how to apply it to the fields mentioned above, especially in biological field."

The authors amend it as follows:

Mortier and Patriarche [2] showed that PbF2 nanoparticles could be produced by dissolving the amorphous GeO2-PbO phase of GCs with HF. Unfortunately, the corrosion between GeO2-PbO and HF can hardly be complete so that the nanoparticle had an amorphous phase as a shell in their experiment. Therefore, no publication has been reported about how to obtain free and pure crystalline nano-particles in aqueous solution from the GC-host and how to apply it to the fields mentioned above.
